# Transformation of a plasmid-free, genital tract isolate of *Chlamydia trachomatis* with a plasmid vector carrying a deletion in CDS6 revealed that this gene regulates inclusion phenotype

**DOI:** 10.1111/2049-632X.12024

**Published:** 2013-02-13

**Authors:** Yibing Wang, Lesley T Cutcliffe, Rachel J Skilton, Kenneth Persson, Carina Bjartling, Ian N Clarke

**Affiliations:** 1 Faculty of Medicine, Molecular Microbiology Group, University of Southampton, Southampton General HospitalSouthampton, UK; 2 Department of Laboratory Medicine, Clinical Microbiology, Institute of Clinical Sciences, Malmo University HospitalMalmo, Sweden; 3 Department of Obstetrics and Gynaecology, Institute of Clinical Sciences, Malmo University HospitalMalmo, Sweden

**Keywords:** *Chlamydia trachomatis*, plasmid, transformation, deletion, CDS 6, glycogen biosynthesis

## Abstract

The development of a plasmid-based genetic transformation protocol for *Chlamydia trachomatis* provides the basis for the detailed investigation of the function of the chlamydial plasmid and its individual genes or coding sequences (CDS). In this study we constructed a plasmid vector with CDS6 deleted (pCDS6KO) from the original *Escherichia coli/C. trachomatis* shuttle vector pGFP::SW2. pCDS6KO was transformed into a clinical isolate of *C. trachomatis* from Sweden that is plasmid-free (*C. trachomatis* SWFP–). Penicillin-resistant transformants expressing the green fluorescent protein were selected. These transformants did not stain with iodine, indicating that this property is regulated by CDS6 or its gene product. In addition, mature inclusions of *C. trachomatis* SWFP– transformed by pCDS6KO displayed an identical morphological phenotype to the untransformed plasmid-free recipient host. In this phenotype the morphology of inclusions was altered with the chlamydiae lining the periphery of the inclusion leaving a ‘hole’ in the centre. These green fluorescent inclusions appear ‘doughnut-shaped’ with an empty centre when examined under blue light, giving rise to a characteristic ‘black hole’ phenotype. Our study demonstrates the power of the new genetic system for investigating chlamydial gene function using gene deletion technology.

*Chlamydia trachomatis* is a major human pathogen responsible for a variety of important diseases including trachoma and genital tract infections. It is an obligate intracellular pathogen that replicates within a specialized cytoplasmic structure known as an inclusion and has a unique developmental cycle (Ward, 1983). Cell infection is initiated by the elementary body or EB. EBs are taken up into a specialized vesicle that becomes a mature inclusion within which they differentiate into the larger replicative noninfectious form of the microorganism, the reticulate body or RB. RBs undergo 8–10 divisions within the inclusion and then condense to become EBs (Lambden *et al*., 2006). RBs regulate the inclusion and control of many cellular factors through secretion of proteins from the inclusion into the cell cytoplasm (Kokes & Valdivia, 2012). *Chlamydia trachomatis* isolates are divided into at least 15 serovars which are associated with the different disease pathologies. Serovars A, B and C are associated with blinding trachoma and serovars D–K are responsible for nondisseminating sexually transmitted infections. Thus, these serovars (A–K) infect epithelial cells and are collectively known as the ‘trachoma’ biovar. By contrast, *C. trachomatis* serovars L1, L2 and L3 cause a highly invasive, rare, sexually transmitted disease known as lymphogranuloma venereum (LGV) and thus belong to the LGV biovar. LGV isolates are a popular choice for laboratory work as they usually grow faster although they do have subtly different, less compact inclusion morphology compared with trachoma biovar isolates. These biological differences are also supported by phylogenetic and genome differences, reinforcing the clinical observations that LGV and trachoma isolates can confidently be split into discrete disease entities (Harris *et al*., 2012).

Most isolates of *C. trachomatis* carry a 7.5-kb plasmid (Thomas *et al*., 1997). The plasmid encodes eight coding sequences (CDS) which are arranged so that all but one of the genes are transcribed in the same direction. Assignment of biological function to the eight plasmid genes has been difficult because of the absence of a simple means to manipulate the chlamydial genome (Rockey, 2011). Bioinformatic analyses have indicated that CDS1–3 are probably involved in plasmid replication as CDS1 and 2 are organized into an iteron-type structure regulating the plasmid origin of replication and both sequences share some homology, and are also distantly related to other bacterial recombinases/replication enzymes, indicating that these genes are involved in regulating plasmid replication. CDS3 has homology to the *Escherichia coli DnaB* gene, which encodes an enzyme involved in unwinding DNA during replication (Hatt *et al*., 1988). CDS7 and 8 display sequence homology with other plasmid partition proteins and thus are likely to be essential for plasmid maintenance. Comparison of the phylogenies of plasmids and chromosomes suggests that plasmid evolution remains closely linked to its cognate host chromosome and thus that plasmids and chromosomes are coinherited (Seth-Smith *et al*., 2009).

Recently, we developed a simple transformation protocol based on using the chlamydial plasmid as a vector and calcium chloride (CaCl_2_) treatment of LGV elementary bodies to render them competent to take up plasmid DNA (Wang *et al*., 2011). LGV isolates were selected as they grow faster than trachoma biovars and are easier to transform. We initially used an LGV plasmid and a matched LGV host but we also used a mismatched trachoma plasmid (from the Swedish new variant)/LGV recipient host pair in this work to prove transformation across the biovars was possible.

Our longer term aim is to define the minimal sequences necessary for a shuttle vector by selected deletion. The ability to selectively delete regions of the plasmid also affords the possibility of dissecting further the properties and traits associated with them. A key part of our experimental design was to perform these transformations with plasmid-free isolates, thereby removing the possibility of complementation of the deletions by the endogenous plasmid. A second and important part of our experimental design was to accurately ascribe and match phenotype to genetics, so we considered it essential to use the correctly paired plasmid/recipient host combination to ensure optimal expression of the plasmid-related phenotype.

Naturally occurring plasmid-free *C. trachomatis* are exceedingly rare and only four live isolates have been described (Peterson *et al*., 1990; Persson *et al*., 1996; Farencena *et al*., 1997; Stothard *et al*., 1998). It is possible to cure *Chlamydia* of its plasmid by treatment with Novobiocin (O'Connell & Nicks, 2006). These naturally occurring plasmid-free and plasmid-cured *Chlamydia* all display an unusual inclusion morphology and fail to accumulate glycogen. Studies on natural plasmid-free and plasmid-cured *C. trachomatis* have indicated that the plasmid is a virulence factor and its absence is associated with reduced disease severity (Carlson *et al*., 2008; Olivares-Zavaleta *et al*., 2010; Kari *et al*., 2011; O'Connell *et al*., 2011).

Transformation of plasmid-free *C. trachomatis* L2 with complete wild-type coding sequences (as part of the shuttle vector pGFP::SW2) restored both the ability to accumulate glycogen and wild-type inclusion morphology, proving that these traits are plasmid-encoded (Wang *et al*., 2011). However, our starting point for the current work was to use a genital tract (trachoma biovar) isolate with a matched genital tract plasmid; in this way we planned to optimize expression of the plasmid-associated phenotypes. For this we used a naturally occurring plasmid-free genital tract isolate from Sweden, *C. trachomatis* SWFP–, as a recipient host (Persson *et al*., 1996). We transformed *C. trachomatis* SWFP– using the CaCl_2_-based transformation protocol developed for *C. trachomatis* L2 with the following modifications: an increased amount of *C. trachomatis* SWFP– EB for transformation (plasmid-free strains are sensitive to the CaCl_2_ treatment) and centrifugation (754 ***g*** for 30 min) to initiate cell infection.

As the replication/maintenance proteins encoded by CDS1–3 and 7 and 8 are unlikely to have dual functions, we considered that chlamydial phenotypes would be determined by the other genes. Initially we attempted to delete CDS4 from the shuttle vector pGFP::SW2. However, it has not proven possible to recover *C. trachomatis* transformants when this gene was deleted from the shuttle vector. Thus it appears that CDS4 may have a role in an as yet unknown essential plasmid function.

The next step in our strategy was to attempt deletion of CDS5 and 6; initially we made a construct pCDS5&6KO with both genes deleted from pGFP::SW2 (Wang *et al*., 2011). The deleted region in pGFP::SW2 starts after the CDS4 stop codon (TAA) and ends before the CDS6 stop codon TAG (Fig. 1a). pCDS5&6KO was constructed by replacing the 2452-bp PacI (ORF4) and PsiI (ORF8) fragment in pGFP::SW2 with a 1227-bp PacI–PsiI fragment from the ORF7-8 PCR product using DNA template pGFP::SW2 and primers ORF7_F(*PacI*) (5′-AAAAAATTAATTAAACTAGTTAGACAACTTACTCTAACGTTGGAGTTG-3′) and ORF8_R(PsiI) (5′-GGGGAGGTTTATAAAAAGCTCGTAATATGC-3′). As a result, a 1231-bp DNA fragment (including CDS5 and 6 and their promoters) was deleted from pGFP::SW2, and replaced with a unique SpeI site.

**Fig. 1:**
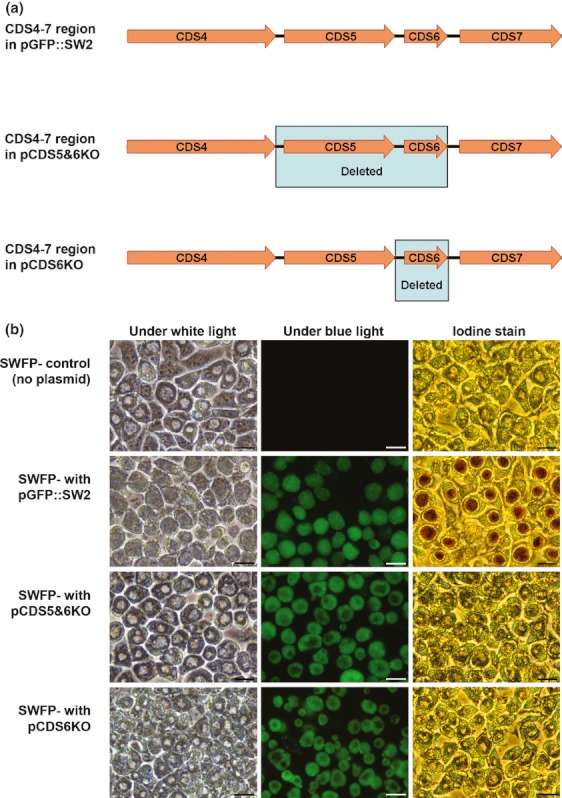
Properties of *Chlamydia trachomatis* SWFP– and its three transformants with pGFP::SW2, pCDS5&6KO and pCDS6KO. (a) Diagrams of pGFP::SW2, pCDS5&6KO and pCDS6KO, highlighting the deleted regions from pGFP::SW2 (in blue boxes). (b) Images of *C. trachomatis* SWFP– and its three transformants 2 days after infection. Left column, images of live McCoy cells infected with different *C. trachomatis* strains under white light (phase contrast); middle column, the same fields under blue light; right column, iodine-stained McCoy cells (methanol-fixed on coverslips) infected with different *C. trachomatis* strains. *Chlamydia trachomatis* SWFP– transformed with pCDS5&6KO or pCDS6KO were not able to accumulate glycogen, and their mature inclusions were morphologically identical to the untransformed recipient parental *C. trachomatis* SWFP– (‘hole’ in the centre). Scale bars = 20 μm.

As CDS6 is the smallest plasmid gene, we also devised a simple strategy to make a construct, pCDS6KO, with this CDS deleted in its entirety from the original plasmid vector pGFP::SW2. The deleted region in pGFP::SW2 starts after CDS5 stop codon TAA and ends before CDS6 stop codon TAG (Fig. 1a). pCDS6KO was constructed by inserting the 860-bp PacI–SpeI fragment from CDS5 PCR product into the PacI–SpeI sites of pCDS5&6KO, so that CDS5 returned to its original location. The CDS5 PCR was performed by using primers ORF5_F(PacI) (5′-CAAGAATCTATTAATTAATAGCAAGCTTGAAAC-3′) and ORF5_R(SpeI) (5′-AAAAAAACTAGTTTAAGCGTTTGTTTGAGGTATTACC-3′), and pGFP::SW2 as DNA template. As a result, a 375-bp DNA fragment (including CDS6 and its promoter) was deleted from pGFP::SW2, and replaced with a unique SpeI site. The PCR regions and the ligation sites of both pCDS5&6KO and pCDS6KO were verified by sequence analysis. All genetic manipulations and containment work was approved under the UK Health and Safety Executive Genetically Modified Organisms (contained use) regulations 2000 notification no. GM57,10.1 entitled ‘Genetic transformation of Chlamydiae’.

We were able reproducibly to recover transformants of *C. trachomatis* SWFP– with pGFP::SW2 and the two new vectors carrying the CDS6 deletion, pCDS5&6KO and pCDS6KO. The properties of transformants were analysed by phase contrast microscopy, green fluorescence protein (GFP) imaging and iodine staining (Fig. 1b). All three transformants expressed GFP. However, the distribution of green fluorescence was different. In *C. trachomatis* SWFP– transformed with pCDS5&6KO or pCDS6KO, green fluorescence was concentrated in the perimeter of the inclusions, leaving a ‘black hole’ in the centre (Fig. 1b). The phase contrast images were identical to the untransformed plasmid-free parental strain SWFP–. Both of the transformants were iodine stain-negative (Fig. 1B). Transformed *C. trachomatis* SWFP– with pGFP::SW2 displayed normal inclusion morphology and was iodine stain-positive, indicating the accumulation of glycogen (Fig. 1b).

Inclusions of *C. trachomatis* SWFP– transformed with CDS6 deletion vectors could not be stained with iodine, indicating that glycogen accumulation was dependent on the presence of CDS6; and the inclusion morphology was identical to the untransformed recipient parental *C. trachomatis* SWFP– proving that CDS6 was essential for maintaining the normal inclusion morphology. Thus, we have conclusively shown that the *C.trachomatis* plasmid CDS6 is responsible for the plasmid-associated phenotype of glycogen accumulation/biosynthesis. Transcriptional analyses of the CDS6 region have not indicated the presence of highly abundant noncoding RNAs characteristic of other regions of the plasmid (Ricci *et al*., 1995; Albrecht *et al*., 2010). Further molecular/genetic analysis of CDS6 is required to elucidate the molecular mechanism(s) by which it or its encoded polypeptide regulates glycogen biosynthesis and influences inclusion morphology. A simple explanation of the ‘black hole’ inclusion morphology could be that the growth rate of the CDS6 knockout mutant(s) is reduced at the mid stage of the developmental cycle, generating a distinctive inclusion whose membrane grows normally and would yield doughnut-shaped inclusions with replicating RBs at the periphery. This is consistent with the contact-dependent development model of intracellular development (Wilson *et al*., 2006, 2009; Peters *et al*., 2007). It is possible that the protein encoded by CDS6 is also the factor responsible for conferring the virulence properties associated with the *C. trachomatis* plasmid. Future work will include assessing the properties of the *C. trachomatis* transformants described here in animal models of infection.
